# The relationship and difference between delay detection ability and judgment of sense of agency

**DOI:** 10.1371/journal.pone.0219222

**Published:** 2019-07-09

**Authors:** Michihiro Osumi, Satoshi Nobusako, Takuro Zama, Naho Yokotani, Sotaro Shimada, Takaki Maeda, Shu Morioka

**Affiliations:** 1 Neurorehabilitation Research Center, Kio University, Nara, Japan; 2 Department of Electronics and Bioinformatics School of Science and Technology, Meiji University, Kawasaki, Japan; 3 Department of Neuropsychiatry, Keio University School of Medicine, Tokyo, Japan; University of Rome, ITALY

## Abstract

Judgment of agency involves the comparison of motor intention and proprioceptive/visual feedback, in addition to a range of cognitive factors. However, few studies have experimentally examined the differences or correlations between delay detection ability and judgment of agency. Thus, the present study investigated the relationship between delay detection ability and agency judgment using the delay detection task and the agency attribution task. Fifty-eight participants performed the delay detection and agency attribution tasks, and the time windows of each measure were analyzed using logistic curve fitting. The results revealed that the time window of judgment of agency was significantly longer than that of delay detection, and there was a slight correlation between the time windows in each task. The results supported a two-step model of agency, suggesting that judgment of agency involved not only comparison of multisensory information but also several cognitive factors. The study firstly revealed the model in psychophysical experiments.

## Introduction

Sense of agency (SoA) is the subjective feeling of initiating and controlling one’s own action and external events [1–3]. For example, SoA may be experienced as a person’s sense that they are causing something to move, or that they are generating a certain thought in their stream of consciousness [2,3]. The feeling of being an agent of an action can be implicitly represented by comparing motor intention and proprioceptive/visual feedback information (i.e., sensorimotor integration) [4]. When incongruencies between these indicators are noticed (e.g., a mismatch between proprioceptive/visual feedback information and motor intention), several odd sensations are evoked during an action. We previously demonstrated that experimentally manipulating the time delay between motor intention and visual feedback information evoked odd sensations, for example, limb heaviness, distorted limb ownership in participants [5]. However, “judgment” of agency would be expected to be only partially distorted despite noticing the incongruence between motor intention and visual feedback information. For example, people could detect the temporal discrepancy between motor intention and visual feedback of approximately 250 msec [5], but the temporal discrepancy may be insufficient to attenuate their judgment of agency [6]. These findings suggest that the time window of delay detection and judgment of agency differ, although the delay detection and the judgment of agency share a common mechanism. Regarding the underlying mechanisms, SoA is thought to be modulated via an optimal “cue integration” process [7]. Therefore, cognitive processes such as thoughts and beliefs are thought to influence the relationship between delay detection and the judgment of agency [4,8]. Additionally, voluntary action selection [9] and social context [10] can modulate SoA. A previous study reported a weaker relationship between time delay and SoA using structural equation modeling [11], suggesting that the integration of actions and effects is only one of multiple cues that contribute to SoA. However, none of the previous reports have directly investigated the correlations and differences between time windows of delay detection and judgment of agency within the same subject. The present study aimed to experimentally reveal these relationships using a psychophysical experimental paradigm. Considering the previous findings discussed above, we predicted that the comparing motor intention and feedback information would be only one of multiple factors that contribute to a sense of agency. Thus, we hypothesized that there would be a partial correlation between the time windows of visual-motor delay detection and judgment of agency, but that the effect size would be small.

## Methods

### Participants

We used convenience sampling to recruit 58 healthy subjects (22 males, 36 females; mean age, 21.05 years; SD, 1.09). None of the subjects had any diseases affecting their color perception or visual acuity. The study protocol was in accordance with the Declaration of Helsinki. All participants were informed at the start of the study that they could discontinue participation at any time during the experiments. We explained the details of the experimental procedure, but not the purpose of the experiment, to avoid bias in the results. Participants provided written informed consent before participating. This study was approved by the ethics committee of Kio University Health Science Graduate School (approval number: H29-26).

### Judgments of agency

**Fig 1:**
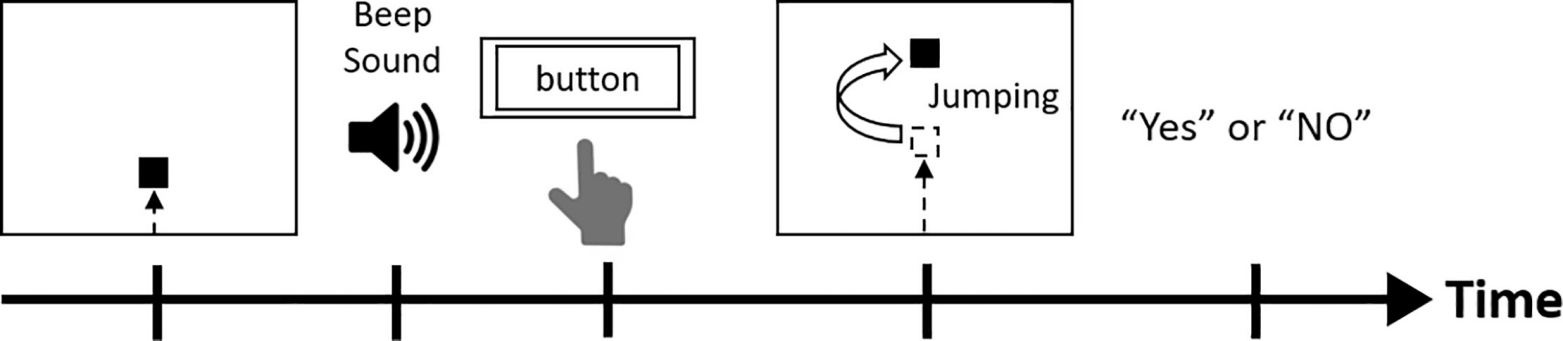
Example of a trial in the agency attribution task. A square, which moved directly upward on the monitor, jumped upward when the subject pressed a button. A time delay was inserted between the button press and the jumping movement of the object. Participants then reported whether they felt that they had caused the square to jump upward in each time delay condition.

The agency attribution task [12, 13] was conducted to quantify the time window of SoA judgment for each participant (Fig 1). The experimental stimuli were presented on a 14-in computer monitor. A white 5-mm square appeared from the bottom of a black screen and moved directly upward at a uniform speed (22 mm/sec). Participants were instructed to press a button as quickly as possible with their dominant index finger when they heard a beep sound. When participants pressed the button, the square jumped 35 mm upward at a delay that was randomly selected from 11 delay conditions (0, 100, 200, 300, 400, 500, 600, 700, 800, 900, and 1000 msec) using Visual Basic software (Microsoft Corp., Redmond, WA, USA). Participants were required to verbally report whether they felt that the square’s jumping was caused by their own preceding action. A “Yes” response meant that the participants attributed the jumping of the square to their button press (i.e., they felt a SoA during the action). A “No” response meant that the participant did not feel that their button press had caused the jumping of the square. We encouraged subjects to judge the sense of agency rather than the time simultaneity in the agency attribution task. Each delay condition was conducted 10 times (i.e., 11 conditions × 10 times = 110 trials). After 10 trials, we confirmed whether subjects’ responses were based on judgments of agency and not simultaneity. In addition to these trials, participants also experienced “event prior to action (EPA)” trials, in which the square jumped when the beep occurred instead of when the key was pressed [12, 13]. There were three EPA conditions, in which the square jumped 100 msec before the beep, at the time of the beep, or 100 msec after the beep. Participants were again instructed to report whether they felt a SoA. Each EPA condition was run 10 times (i.e., 3 conditions × 10 times = 30 trials). We conducted the EPA trials as dummy stimulation. If participants responded “Yes”, their responses were considered to not be based on agency judgment. The EPA trials were mixed into the agency attribution task at random. Thus, there was a total of 140 trials. As described above, we conducted different tasks from the below-mentioned delay detection task to quantify the time window of agency judgments, to prevent interference with each task.

### Delay detection task

**Fig 2:**
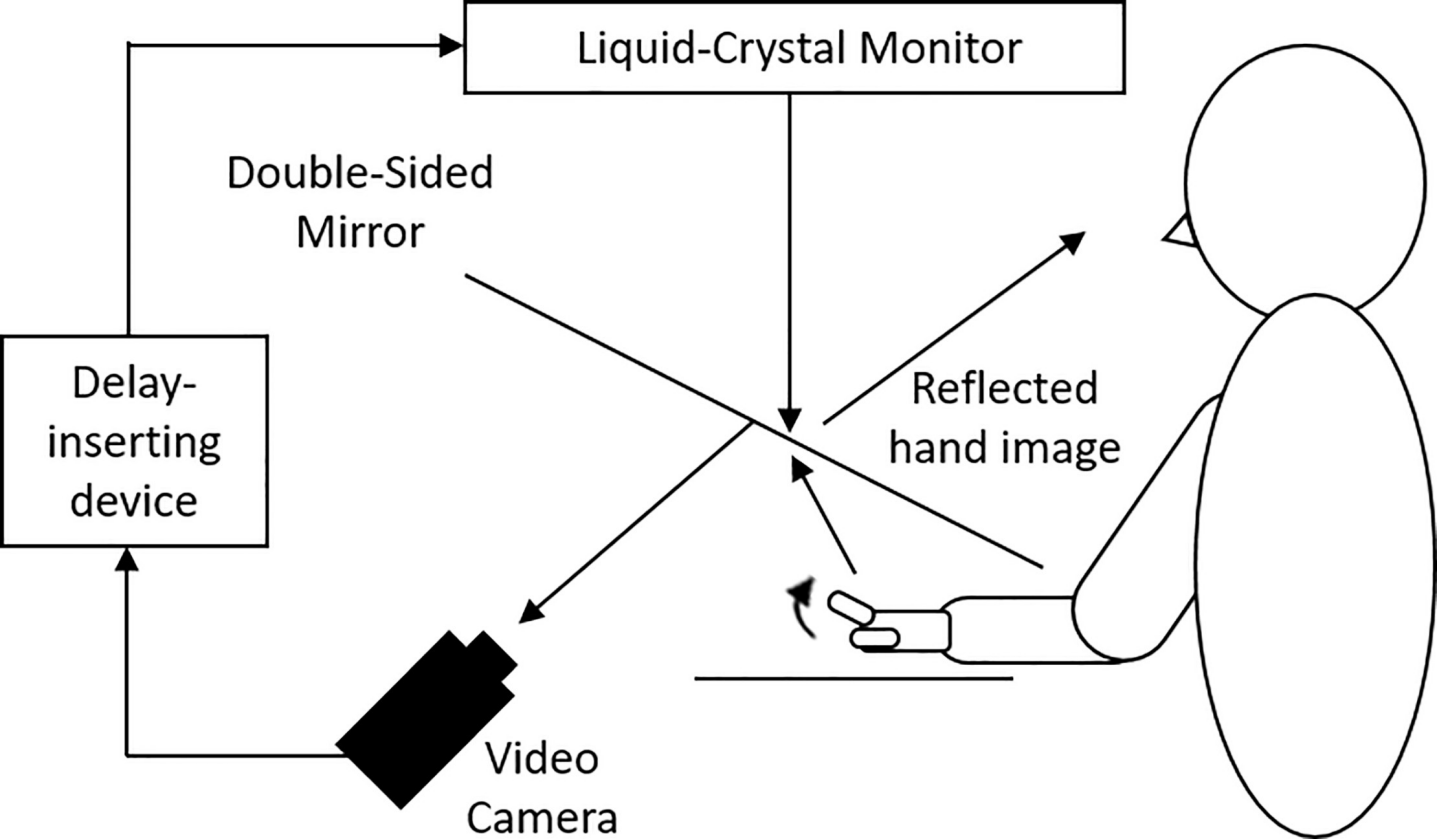
Demonstration of the experimental setup in the delay detection task. Participants watched an image of their moving hand that was delayed following their actual movement. They reported whether the visual feedback of the finger movement was exactly synchronized with their hand movement execution after each trial.

We used our experimental system (Fig 2) to quantify participants’ delay detection ability as a measure of sensorimotor function. The present experimental setup could systematically delay the time between movement execution and visual feedback, with an experimental system similar to that used in previous studies [5,14]. Participants were not able to directly view their hand, which was placed under a double-sided tilted mirror. The reflected image of their hand in the double-sided mirror was filmed with a video camera (FDR-AXP35, Sony, Tokyo, Japan). The filmed hand image was sent to a liquid-crystal display monitor (LMD-A240, Sony) through a video delay device (EDS-3306, FOR-A YEM ELETEX, Tokyo, Japan). Finally, the hand images from the monitor were projected onto the double-sided mirror, enabling the participants to observe the image of their own hand reflected in the mirror without seeing their actual hand. The angle of the mirror was finely adjusted before the experiment, so that the reflected hand image was viewed from the participant’s perspective as if it were placed horizontally on the table. Visual feedback delay was introduced using a hardware device (EDS3305, ELETEX, Osaka, Japan) connected between the video camera and the monitor. Eleven delay conditions (0, 100, 200, 300, 400, 500, 600, 700, 800, 900, and 1000 msec delay) were tested. The intrinsic delay of the visual feedback in this experimental setting was approximately 33.71 msec, as measured by a time lag check device (EDD-5200, FOR-A YEM ELETEX, Tokyo, Japan). For simplicity, we refer to these delay conditions as: 0, 100, 200, 300, 400, 500, 600, 700, 800, 900, and 1000 msec. In the experiment, participants were asked to move their index finger just once, and to judge whether the visual feedback of finger movement (i.e., the reflected finger image) was exactly synchronized with their finger movement execution after each trial. Ten trials were conducted for each delay condition. Thus, the experiment consisted of 110 trials. The order of the delay conditions was randomized.

### Data analysis

#### Analyzing time window of “SoA attenuation”

In the agency attribution task, the “No” response probability for each delay condition (0, 100, 200, 300, 400, 500, 600, 700, 800, 900, and 1000 msec), excluding the response data in EPA trials, was calculated for each subject. Positive values of the “No” response probability indicated lower SoA in relation to the square. Logistic curves were fitted to the “No” response probability in the agency attribution task using the following formula [14,15]: where t is the delay time and *P(t)* is the probability of “No” response, *a* indicates the steepness of the fitted curve, and *t*_*PSE*_ indicates the observer’s point of subjective equality (PSE), which is the delay time at which the probabilities of “Yes” and “No” judgment are equal (50%). The fitting was performed using a nonlinear least squares method (a trust-region algorithm), which was provided by the Curve Fitting toolbox in Matlab 7.5 (MathWorks, MA, USA).

$$P(t)=\frac{1}{1+exp(-a(t-t_{PSE}))}$$

#### Analyzing “delay detection” time windows

In the delay detection task, the asynchrony judgment probability for each delay condition (0, 100, 200, 300, 400, 500, 600, 700, 800, 900, and 1000 msec) was then calculated for each subject. Positive values of the asynchrony judgment probability indicated a higher ability of delay detection (i.e., better sensorimotor function). Logistic curves were also fitted to subjects’ responses in the asynchrony judgment task on the basis of the formula described above [14,15]: where t is the visual feedback delay length and P(t) is the probability of making an asynchrony judgment, *a* indicates the steepness of the fitted curve, and *t*_*PSE*_ indicates the observer’s PSE, which is the delay length at which the probabilities of synchrony and asynchrony judgment are equal (50%).

#### Analyzing the difference and relationship between delay detection ability and judgment of agency

To clarify the difference of the time windows between sensorimotor function and SoA judgment, we compared the PSE of delay detection with the PSE of SoA attenuation using a t-test. To clarify the relationships between these measures, we analyzed the correlation between the PSE of delay detection and the PSE of SoA attenuation using Pearson’s correlation coefficients.

Effect sizes (*r* values) were calculated by dividing the test statistic by the square root of the number of observations; *r* > .5 generally indicates a large effect size, *r* > .3 indicates a medium effect size, and *r* > .1 indicates a small effect size [16]. The significance level was set at *p* < .05. SPSS ver. 20.0 (IBM Corp., Armonk, NY, USA) was used for statistical analysis.

## Results

**Fig 3:**
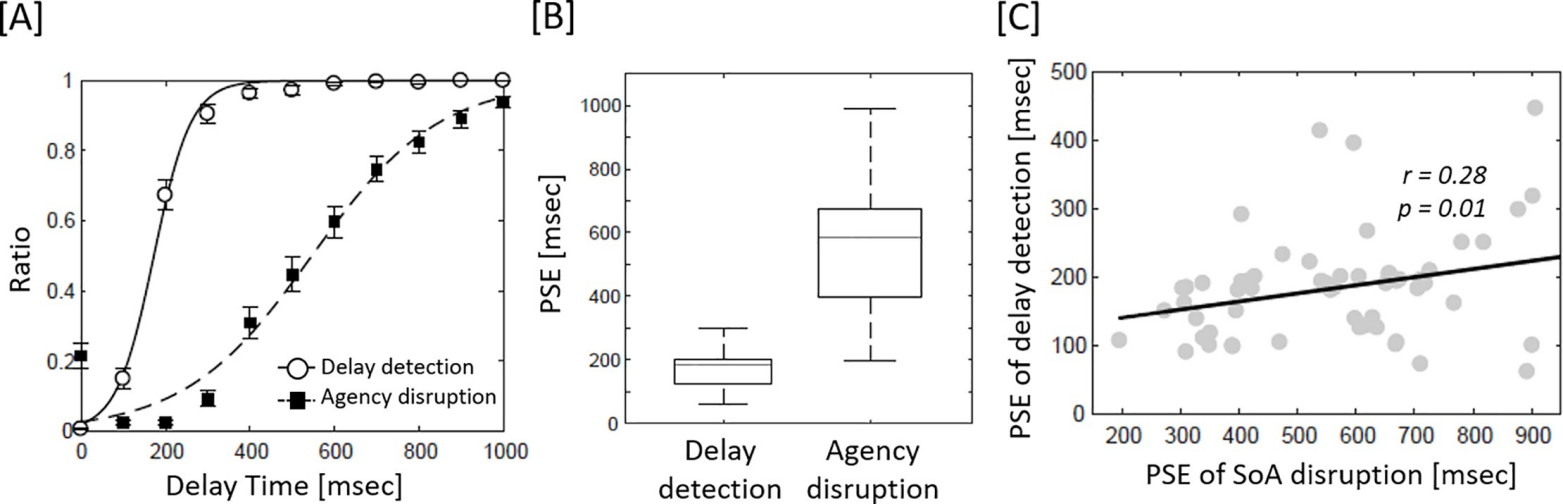
[A] Delay detection curve (solid line) and ratio (white circle), and agency attenuation curve (dotted line) and ratio (black square) are shown in each delay condition. Error bars indicate ± SE. [B] Significant difference in the point of subjective equality (PSE) between delay detection and agency attenuation (*p* < .05). [C] Significant positive correlation between PSE of delay detection and PSE of agency attenuation (*p* < .05).

Fitted curves for delay detection and agency attenuation are shown in Fig 3. The slopes of the fitted curves were discernibly different, and PSE for delay detection was significantly shorter than that for agency attenuation (delay detection: mean 183.94 ± 78.31 SD, agency attenuation: 565.92 ± 190.64 msec, *t* = −15.806, *r* = .90, *p* < .0001, Fig 3), indicating that the timing of delay detection was significantly faster than that of agency attenuation. In addition, the PSE of delay detection was significantly correlated with that of agency attenuation (*t* = 2.25, *r* = .28, *r*^*2*^ = .083, *p* = .014, Fig 3). Post-hoc power calculation of η^2^ revealed a value of 0.83. However, the r value indicated that the effect size was relatively small [16], indicating that the relationship between delay detection ability and SoA was statistically significant, but the relationship was not strong. It should be noted that participants gave very few yes responses in the EPA condition: −100 msec; 0.21 ± 0.45 (average ratio ± SD), 0 msec; 0.28 ± 0.45, + 100 msec; 0.39 ± 0.61, indicating that they conducted the agency attribution task correctly.

## Discussion

In the present study, we investigated the difference in time windows between delay detection and judgment of agency. The results revealed that the time window of agency attenuation was longer than that of delay detection, indicating a difference in the relationship between them. The PSE of delay detection reflects the ability to integrate motor intention and visual feedback information (i.e., sensorimotor integration ability) [14]. In one previous study, patients suffering from sensorimotor dysfunction, such as coordination disorder, were reported to be unable to detect time delay between motor intention and visual feedback information [17]. In addition, several neuroscientific studies have suggested that delay detection is related to activity in the supplemental motor area and parietal area, which is related to sensorimotor integration [18,19]. The judgment of agency also involves sensorimotor integration, but its cognitive processes differ from delay detection, and are influenced by several additional factors, including intention and thoughts. For example, intentional effort [20] and goals [21] predictability [22] are reported to influence the explicit SoA. In addition, a previous study [23] reported the different time window of agency disruption compared with the present results, in which healthy participants were asked to judge SoA of their own hand, rather than a square object. Embodiment of a participants’ own hand would be likely to differ from that of an external object, and this suggestion is supported by experiments using the rubber hand illusion [24]. Thus, we propose that the appearance of the body may influence the judgment of agency. These previous findings suggest that the cognitive processes underlying judgment of agency might result in a longer time window compared with delay detection, in accord with the present results. Several recent studies have reported the effects of rehabilitation with virtual reality systems to elicit an illusory sensation: *“I felt as if I could control the movements of the virtual limb”* [25–27]. To enhance the SoA, a virtual reality system can present the subject with virtual-visual feedback corresponding to their motor intention. However, the time between motor intention and visual feedback is often delayed due to technical constraints. The results of the present study indicate that the time window of agency attenuation was longer than that of delay detection. Thus, the time delay between motor intention and visual feedback may not be considered a problem for eliciting SoA, even though the subject may detect the time delay. On the other words, small delays inherent to virtual reality setups may not impede SoA. Previous findings suggest that virtual reality could enhance SoA and improve clinical symptoms [27,28].

The results of the correlation analysis in the present study revealed that the PSE of delay detection was correlated with the PSE of agency attenuation, indicating that sensorimotor integration ability was closely related to the explicit SoA. A previous study also reported that patients with schizophrenia exhibiting an abnormal explicit SoA simultaneously exhibited impaired sensorimotor integration [29], suggesting a close relationship between sensorimotor integration ability and explicit SoA from a clinical perspective. To the best of our knowledge, the current study is the first to successfully reveal the relationship between sensorimotor integration and SoA with an experimental paradigm involving the delay detection task and the agency attribution task. However, an effect size of *r* = .28 is considered relatively weak in correlation analysis. This modest effect size suggests that delay detection and judgment of agency are derived, at least in part, from different mechanisms. One previous study reported patients with impaired sensorimotor integration ability but preserved judgment of agency [30]. Taken together with previous findings [4, 20, 21, 22], the current results suggested that explicit SoA is based on sensorimotor integration, but may also be affected by cognitive processes such as thoughts, beliefs, intentions, goals, and predictability. In future studies, we intend to investigate which factors modulate explicit SoA.

## Conclusion

To the best of our knowledge, the present study is the first to reveal differences and correlations of time windows between delay detection and explicit SoA attenuation within the same subject. Although sensorimotor incongruence induces both odd sensations and SoA attenuation, their time windows were significantly different and the effect size of correlation analysis was low. These results suggested that several cognitive factors attenuate the relationship between delay detection and judgment of agency. We conclude that the comparator model is only one of multiple factors that contribute to the sense of agency.

## Limitations

The current study involved several limitations that should be considered. First, we were not able to control the sensory modality between the agency attribution task (motor and visual) and the delay detection task (motor/proprioceptive and visual). However, considering previous reports [31] of correlations between audio-visual, audio-tactile, and visuo-tactile simultaneity judgments, the difference in sensory modality between the agency attribution task and the delay detection task was not likely to have been a serious problem. Second, we did not objectively assess variations in color perception or the visual acuity of participants.

## Supporting information

S1 File Supporting information files. (XLSX)
